# Use of the ORBEYE™ Exoscope for the Treatment of Presumed Postoperative Cholesteatoma of the External Auditory Canal: A Case Report

**DOI:** 10.3390/jcm15218625

**Published:** 2026-11-05

**Authors:** Carlotta Liberale, Arianna Burato, Livio Presutti, Giuseppe Mercante, Giulia Molinari

**Affiliations:** 1 Department of Otolaryngology-Head and Neck Surgery, University Hospital of Modena, Azienda Ospedaliero-Universitaria di Modena, 41125 Modena, Italy; 2 Otolaryngology and Audiology Unit, IRCCS Azienda Ospedaliero-Universitaria di Bologna, 40138 Bologna, Italy; 3 Department of Medical and Surgical Sciences (DIMEC), University of Bologna, 40138 Bologna, Italy

**Keywords:** cholesteatoma, external auditory canal, otologic surgical procedures, exoscope, ORBEYE

## Abstract

**Background/Objectives**: To describe the technical feasibility of fully exoscopic management of an unusually extensive external auditory canal cholesteatoma (EACC). **Methods**: We report a 26-year-old man with advanced right EACC and a history of ipsilateral myringoplasty approximately 15 years previously; a postoperative origin was suspected but could not be established. Clinical, radiological, surgical, and follow-up findings are described. **Results**: Imaging revealed extensive erosion of the superior EAC and adjacent temporal bone with mass effect on the right temporal lobe. The ORBEYE™ 4K three-dimensional exoscope was used throughout the operation, including dissection along exposed dura and hearing-preserving reconstruction. An intraoperative cerebrospinal fluid leak occurred at the middle cranial fossa dura and was controlled during surgery, without persistent postoperative leakage. The patient was discharged on postoperative day 4. Based on the reported four-frequency pure-tone averages, the calculated air–bone gap decreased from 18.5 to 8.5 dB HL. Diffusion-weighted MRI at 18 months showed no evidence of residual or recurrent disease, and clinical examination at 24 months showed a patent, epithelialized canal. **Conclusions**: This case illustrates the technical feasibility of a fully exoscopic, hearing-preserving approach to complex EACC. Comparative studies are required before conclusions about superiority or generalizable safety relative to conventional visualization can be drawn.

## 1. Introduction

External auditory canal cholesteatoma (EACC) is a rare condition [1]. It should be differentiated from keratosis obturans (KO), squamous cell carcinoma (SCC), and malignant otitis externa (MOE). Its incidence has been reported as approximately 1 in 1000 otologic patients [2]. EACC may be primary (idiopathic) or secondary to congenital, post-traumatic, postoperative, or inflammatory factors [3]. Naim et al. [4] described four progressive histopathological stages: (I) focal epithelial hyperplasia; (II) inflammation and periostitis; (III) keratin accumulation with erosion of the bony canal; and (IV) erosion of adjacent anatomical structures.

Secondary and iatrogenic EACCs may develop as a consequence of epithelial entrapment or altered migration of the external auditory canal skin following trauma or previous otologic surgery. Clinical presentation may be nonspecific and can include otalgia, otorrhea, aural fullness, and conductive hearing loss. Because symptoms may remain limited until significant bone erosion has occurred, imaging plays an important role in assessing disease extension. High-resolution computed tomography (CT) is particularly useful for identifying bony erosion and involvement of the mastoid, temporomandibular joint, facial nerve canal, and cranial fossae, whereas diffusion-weighted magnetic resonance imaging (MRI) can support the diagnosis of cholesteatoma and define its relationship with intracranial structures [1,3].

The treatment of EACC depends on its extent. Limited lesions may be managed by local debridement and/or canalplasty, whereas advanced disease with mastoid, temporomandibular joint, or dural involvement may require mastoidectomy or more extensive temporal bone surgery [3]. In these cases, complete removal of the cholesteatomatous matrix must be balanced against preservation of functional structures, particularly the tympanic membrane, ossicular chain, facial nerve, and dura. Hn et al. proposed a clinically oriented staging system in which stage III disease is characterized by bone erosion with extension to adjacent structures, with or without complications [3].

The present report describes the surgical management of an unusually extensive EACC using the ORBEYE™ exoscope (Olympus, Tokyo, Japan), which provides stereoscopic 4K images on a 55-inch monitor [5]. Exoscopic visualization has previously been described in otologic and neurotologic surgery; therefore, use of an exoscope alone does not constitute the novelty of this report. Published reports and comparisons suggest potential technical and educational advantages, but available evidence does not establish consistent clinical superiority over conventional microscopy [5,6,7,8].

Unlike conventional binocular microscopy, the exoscope allows the surgeon to operate while viewing a high-definition stereoscopic image on an external monitor, potentially providing greater freedom in camera positioning, improved ergonomics, and a shared operative view for surgeons, assistants, and trainees. Recent studies and systematic reviews have reported encouraging results for 3D exoscopic visualization in otologic and lateral skull base procedures, although evidence remains limited and heterogeneous [6,7,8].

The specific contribution of this case is the application of a fully exoscopic technique to EACC with extensive superior temporal bone destruction, exposure of middle and posterior cranial fossa dura, a difficult superior extension, and reconstruction intended to preserve middle-ear anatomy and hearing. We describe the operative steps, intraoperative dural event, reconstructive strategy, and follow-up while distinguishing observations in this patient from potential advantages reported elsewhere.

## 2. Materials and Methods

### 2.1. Patient and Diagnostic Work-Up

Written informed consent was obtained from the patient for preparation and publication of this case report and the accompanying images. A 26-year-old man had undergone right myringoplasty through a retroauricular approach approximately 15 years earlier for chronic perforated otitis media with recurrent exacerbations. At a subsequent otolaryngology visit, he reported right-sided aural fullness and hearing loss. Otoendoscopy demonstrated subtotal stenosis of the right EAC with a nonpulsatile bulge of its superior wall. Pure-tone audiometry showed mild right-sided conductive hearing loss. Facial nerve function was normal (House–Brackmann grade I). A postoperative origin was considered because of the history of ipsilateral surgery, but this temporal association cannot establish causality. The operative report from the earlier procedure was unavailable; consequently, the grafting technique, tympanomeatal flap elevation and repositioning, management of EAC skin, and any postoperative canal stenosis or epithelial entrapment could not be verified.

**Figure 1:**
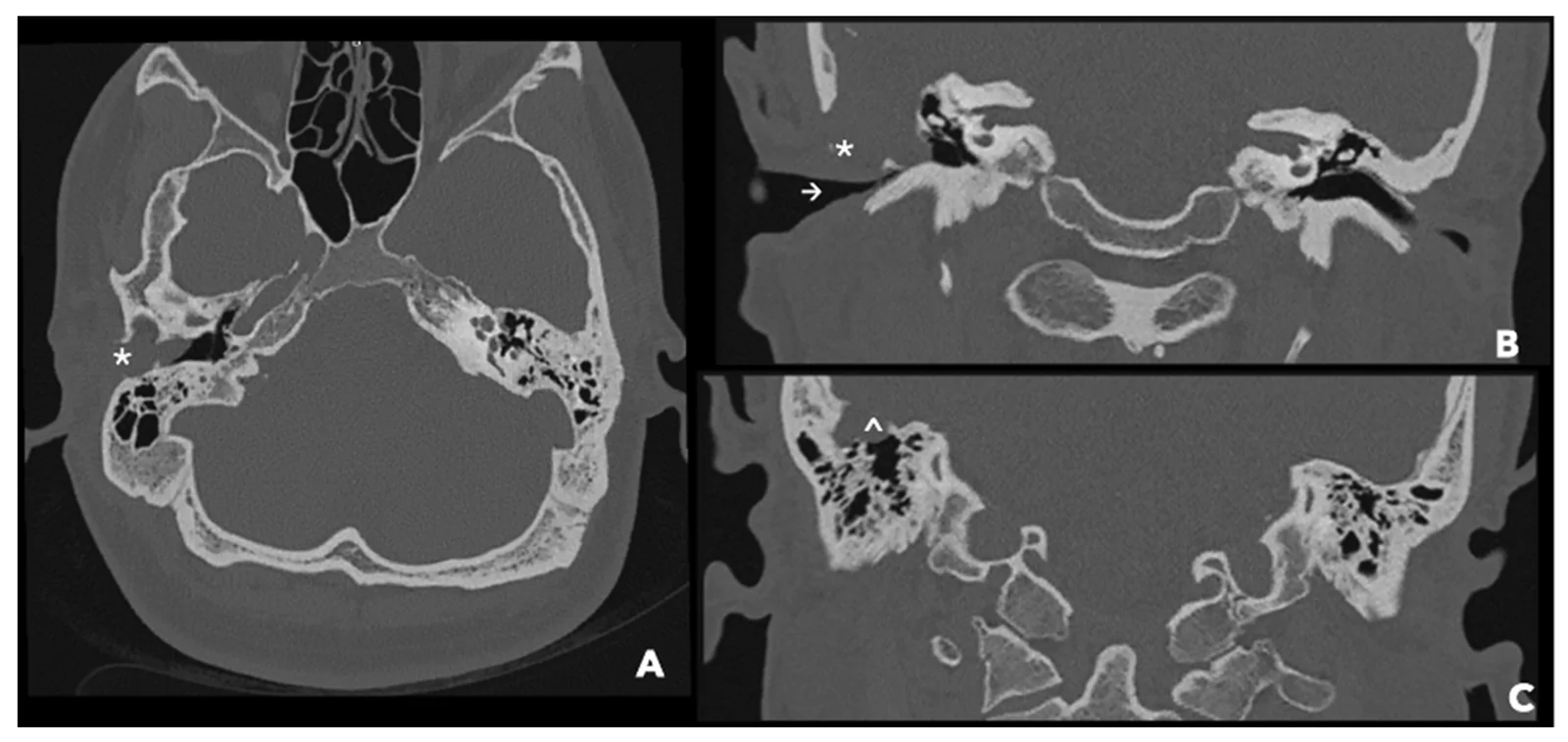
CT of the ear and petrous bones with contrast medium. (**A**) Axial section showing a discontinuity at the level of the temporal bone (*). (**B**) Coronal scan revealing hypodense tissue eroding the lateral aspect of the temporal bone (*) and remodeling the profile of the roof of the EAC, with a reduction in the caliber of the EAC itself (→). (**C**) Coronal scan detailing erosion of the tegmen antri (^) caused by the cholesteatoma.

Temporal bone computed tomography (CT) demonstrated a substantial defect in the superior bony wall of the right EAC, with adjacent soft-tissue attenuation and erosion of the temporal bone and tegmen antri (Figure 1). The imaging supported extension beyond the confines of the EAC; bone erosion should be distinguished from direct invasion of adjacent soft-tissue or intracranial structures.

**Figure 2:**
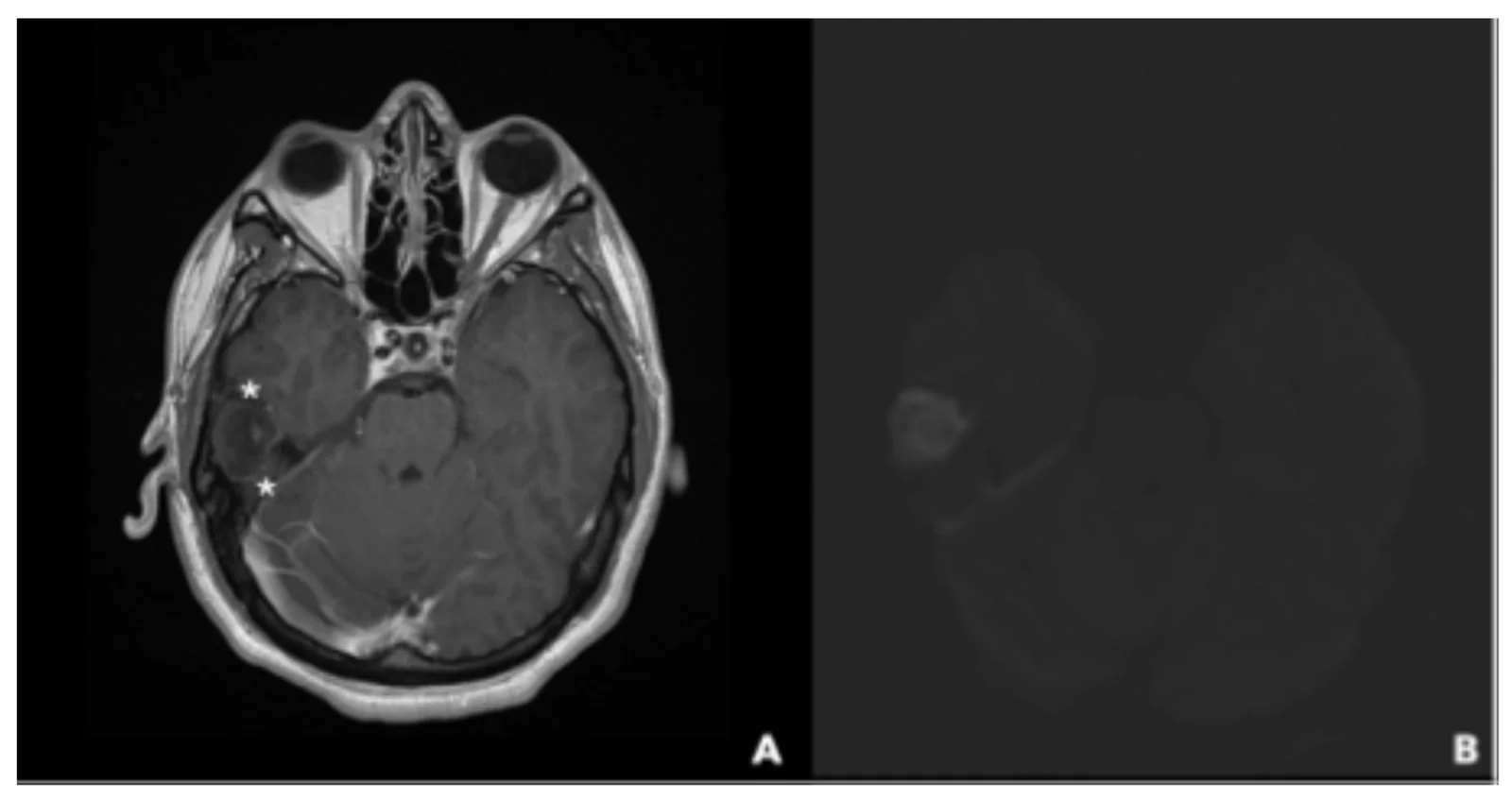
MRI of the brain and temporal bones. (**A**) Axial postcontrast T1-weighted image showing a nonenhancing lesion with mass effect on the right temporal lobe (*). (**B**) Diffusion-weighted image showing restricted diffusion compatible with cholesteatoma.

Magnetic resonance imaging (MRI) demonstrated a nonenhancing lesion with restricted diffusion on diffusion-weighted imaging (DWI), supporting a radiological diagnosis of cholesteatoma. Its superior extension produced an impression on the right temporal lobe (Figure 2). Mass effect on the temporal lobe does not, by itself, demonstrate parenchymal invasion.

Taken together, the findings were consistent with advanced EACC extending outside the bony external canal. Erosion of adjacent structures corresponds to stage IV in the histopathological classification of Naim et al. [4]. According to the clinically oriented system of Hn et al., the lesion was classified as stage IIIa, reflecting extension beyond the EAC with no documented preoperative complication [3].

The patient was then referred for surgical excision of the suspected cholesteatoma of the right EAC via a retroauricular approach using the ORBEYE™ exoscope.

### 2.2. Surgical Equipment and Exoscopic Setting

The procedure was performed under ORBEYE™ 4K three-dimensional (3D) exoscopic visualization (Olympus, Tokyo, Japan). The system displayed the operative field on a 55-inch 4K stereoscopic monitor in front of the primary surgeon; the surgeon and assistant wore polarized 3D glasses and viewed the same operative image. The primary surgeon was seated facing the operative field, while the assistant stood on the patient’s right side. The exoscope optical head was positioned above the surgical field and connected to the display system. Surgical instruments were arranged on a sterile instrument table adjacent to the operating site, allowing access to the operative field under continuous exoscopic visualization. Camera direction, magnification, and focus were adjusted during mastoid drilling, dural dissection, inspection of the superior temporal extension, and canal-wall reconstruction.

The displayed magnification and working-distance settings were retrospectively reviewed from the system monitor. During incision and musculoperiosteal flap elevation, magnification was ×1.3 with a displayed distance of 457 mm; during initial mastoidectomy, ×1.6 at 359 mm. During removal of cholesteatoma from adjacent bony structures, magnification ranged from ×1.9 to ×2.2 (228–269 mm). For matrix dissection within the EAC, magnification was increased to ×3.0 (280 mm). During dissection along the temporal squama, magnification ranged from ×2.6 to ×3.2 (241–363 mm). The region adjacent to the temporomandibular joint (TMJ) was inspected at ×2.8 (320 mm). During preparation of temporalis muscle and tragal cartilage and reconstruction of the EAC wall, magnification was reduced to ×1.1 (approximately 550 mm). These values represent the settings displayed by the system, rather than independently measured distances.

The exoscopic viewing direction was adjusted according to the anatomical region. Initially, the optical head was positioned nearly perpendicular to the mastoid plane, along the EAC axis, to visualize the retroauricular approach and flap elevation. During cortical mastoidectomy and drilling along the temporal squama and tegmen, the optical head was tilted in inferior-to-superior and posterior-to-anterior directions to inspect the superior bony margins and exposed middle cranial fossa dura. For the anterior extension adjacent to the TMJ, the camera was oriented from a posterosuperior position toward the anteroinferior surgical field. Repositioning of the optical head and adjustment of focus were required to inspect recesses while limiting obstruction by surgical instruments. Numerical camera angles were not recorded; the directions above are qualitative descriptions of the operative views.

The conventional operating microscope was not used. Quantitative ergonomic outcomes and assistant satisfaction were not assessed using standardized instruments.

The operative duration was 6 h.

### 2.3. Surgical Technique

Under general anesthesia, the patient was placed supine with the head rotated to the left. Facial nerve function was monitored using two-channel intraoperative facial nerve monitoring (Medtronic Xomed, Jacksonville, FL, USA).

A C-shaped retroauricular incision was performed, followed by layer-by-layer dissection and elevation of a large T-shaped musculoperiosteal flap. Temporalis fascia was harvested for reconstruction. An extensive cholesteatomatous matrix was identified at the superior EAC, with erosion of the mastoid cortex, superior canal wall, and tegmen antri and extension into the posterosuperior mastoid region. The matrix was progressively separated from the markedly thinned residual EAC skin. The tympanic annulus was identified and left in position; the tympanic membrane was intact and the middle-ear cavity was not involved. Thus, the disease originated in the EAC but was not anatomically confined to it. Preservation of the intact tympanic membrane and ossicular compartment supported a hearing-preserving strategy.

A wide cortical mastoidectomy exposed the osseous dehiscence occupied by cholesteatoma. Careful dissection progressively exposed the dura of the middle cranial fossa (MCF), the sinodural angle, and the superior aspect of the posterior cranial fossa (PCF) dura. The dura was markedly thinned in several areas. These findings demonstrated extensive bone erosion and dural exposure, but did not establish that a full-thickness dural defect was present before dissection.

During separation of the cholesteatoma matrix from the thinned MCF dura, an intraoperative cerebrospinal fluid (CSF) leak occurred through a pinpoint defect measuring less than 5 mm. The precise contribution of pre-existing dural thinning versus surgical manipulation to the defect could not be determined. The leak was managed with bipolar coagulation followed by direct application of fibrin glue; a separate multilayer dural reconstruction was not performed. The mastoid cavity was subsequently obliterated using temporalis muscle and additional fibrin glue, providing soft-tissue coverage in the operated region. No further CSF egress was seen on direct inspection, including during an intraoperative Valsalva maneuver performed by the anesthesiologist, or before wound closure. This observation documents intraoperative cessation of the leak, not a formally measured watertight seal.

No macroscopic intradural cholesteatoma component or temporal-lobe parenchymal involvement was identified at surgery. The superior component of the cholesteatoma, situated between the temporal squama and the lateral aspect of the temporal lobe, was removed with drilling of the involved temporal bone. A suspected bony sequestrum was removed from the inner temporal squama. The anterior extension reached the region adjacent to the TMJ, without direct involvement of the joint being identified. Grossly complete removal was achieved after inspection of the surgical recesses along the exposed dura and temporal bone.

Tragal cartilage was used to reconstruct the posterosuperior wall of the EAC, with the residual portion of the native canal wall providing structural support for the graft. The cartilage was reshaped using cold-knife incisions and reinforced laterally with bone pâté and fibrin glue. The more distal portion of the EAC was reconstructed using bone pâté, with temporalis muscle fascia placed medially to promote re-epithelialization of the canal skin. This reconstruction was further reinforced laterally with fibrin glue. The mastoid cavity was obliterated using temporalis muscle and fibrin glue. Notably, the temporalis fascia did not serve as an intervening barrier between the reconstructed EAC wall and the muscle used for mastoid obliteration. No additional skin graft, either split-thickness or full-thickness, was required, as the residual canal skin, together with the temporalis fascia graft, provided adequate coverage for re-epithelialization of the reconstructed EAC. The wound was closed in layers, and a Penrose drain was placed. An absorbable gelatin sponge (Spongostan) was positioned within the reconstructed EAC.

## 3. Results

The ORBEYE™ exoscope was used for mastoid drilling, matrix dissection from the EAC skin, exposure of the tegmen and MCF/PCF dura, dissection at the sinodural angle, inspection of the superior temporal extension, and canal-wall reconstruction. A shared stereoscopic view enabled the primary surgeon and assistant to follow the same operative steps. In the surgeons’ qualitative assessment, the movable viewing axis was useful for examining surfaces in different anatomical planes. No conversion to a conventional operating microscope was required. No objective assessment of visualization quality, surgical ergonomics, or team satisfaction was performed.

An intraoperative CSF leak occurred during dissection at the MCF dura and was managed during the operation, with no further leakage observed before closure. There was no clinically apparent persistent postoperative CSF leakage. The patient was discharged on postoperative day 4. No postoperative facial nerve or neurological deficit, wound infection, or hematoma was observed. CT at 6 and 36 h after surgery showed no hemorrhage or other reported intracranial complication. These postoperative observations should be distinguished from the intraoperative dural event.

Final histopathological examination confirmed the diagnosis of cholesteatoma.

At 24 months, otoendoscopy showed a patent, fully epithelialized EAC without recurrent keratin accumulation. Pure-tone audiometry performed two months after surgery showed improvement in air-conduction and bone-conduction thresholds (Table 1). Based on the reported four-frequency air- and bone-conduction pure-tone averages (500, 1000, 2000, and 4000 Hz), the calculated air–bone gap (ABG) decreased from 18.5 to 8.5 dB HL. Non-echo-planar diffusion-weighted MRI at 18 months showed no evidence of residual or recurrent cholesteatoma. Given the extent of the original disease and reconstruction, continued clinical and diffusion-weighted MRI surveillance is warranted.

## 4. Discussion

Primary EACC is frequently described along the canal floor, whereas secondary EACC may have a less typical distribution [3]. The superior location in this case and a remote history of ipsilateral myringoplasty are consistent with a possible postoperative origin, but they do not prove that the original procedure caused epithelial entrapment. A displaced or improperly repositioned tympanomeatal skin flap is one possible mechanism of postoperative EACC [9,10], not an established mechanism in this patient. Accordingly, the lesion is referred to as “presumed postoperative EACC” throughout this report.

The long interval between the original procedure and diagnosis in the present case is clinically relevant. Secondary EACC may develop slowly and remain minimally symptomatic until substantial erosion of the surrounding temporal bone has occurred. Accordingly, a remote history of otologic surgery should remain part of the diagnostic assessment in patients presenting with unexplained EAC stenosis, keratin accumulation, or focal bony erosion.

CT and MRI provided complementary information about the anatomical extent of this lesion. CT demonstrated erosion of the superior EAC, adjacent temporal bone, and tegmen; MRI demonstrated a nonenhancing, diffusion-restricting lesion with mass effect on the right temporal lobe. At surgery, the matrix extended into the mastoid region and superior temporal squama, with exposure and marked thinning of the MCF and superior PCF dura. The adjacent TMJ region was approached by the anterior extension, but direct articular involvement cannot be inferred without specific imaging or operative evidence. Likewise, radiological temporal-lobe impression must not be equated with intraparenchymal extension.

Several staging systems for EACC coexist and should be identified explicitly. Erosion of adjacent anatomical structures corresponds to stage IV according to Naim et al. [4]. Under the clinical classification proposed by Hn et al. [3], the lesion was assigned stage IIIa because it extended to adjacent structures without a documented complication before surgery. The intraoperative CSF leak is reported separately and is not evidence of a preoperative complication.

Canalplasty may be sufficient for limited EACC, but extensive disease often requires mastoidectomy and, in selected cases, more ablative lateral skull base procedures [3]. The choice of resection should account for disease extent, integrity of the middle ear, and the feasibility of preserving useful hearing.

Because the tympanic membrane and functional middle-ear compartment were intact, we favored an anatomy-preserving approach over a more ablative option such as subtotal petrosectomy.

The main technical challenge was to remove disease extending across the EAC, mastoid, tegmen, dura-adjacent recesses, and temporal squama without sacrificing the intact middle-ear compartment. Cortical mastoidectomy, careful separation of matrix from residual EAC skin and exposed dura, drilling of involved temporal bone, and reconstruction of the posterosuperior canal wall were combined to achieve macroscopic clearance while preserving hearing. This anatomical setting—rather than the use of an exoscope in isolation—accounts for the case’s clinical and educational relevance.

Previous studies have described 3D exoscopic visualization for otologic surgery, including comparisons with conventional microscopy and an educational evaluation of observer experience [5,6,7,8]. The exoscope can provide a shared, magnified stereoscopic monitor view and permit changes in camera orientation without the surgeon remaining fixed to microscope oculars. These features may assist communication and teaching. However, published technical or subjective image-quality findings should not be construed as proof of improved clinical outcomes, particularly in the absence of a controlled comparison.

In the present operation, the exoscope was used during drilling adjacent to the tegmen, dissection along thinned and exposed dura, removal of the superior temporal extension, inspection of recesses at the sinodural angle, and EAC reconstruction. Its stereoscopic shared display allowed the surgeon and assistant to view the same operative planes. Any perceived benefit was qualitative; no formal image-quality, operative-time, or ergonomic measurements were collected for comparison. The depth and shape of the surgical field may also challenge camera positioning and instrument clearance, particularly in narrow recesses.

Ergonomics is another possible advantage of monitor-based surgery. Conventional microscopy can require prolonged fixed head and neck posture; comparative work has reported lower ergonomic risk measures during selected 3D exoscopic procedures [11]. However, surgeon comfort and assistant satisfaction were not measured in the present case, and results from other procedures or exoscope platforms cannot be assumed to apply here.

The shared operative image may also have educational value. Tu et al. [8] prospectively assessed observers’ experience with 3D exoscopic versus microscopic otologic surgery and reported perceived benefits for selected educational outcomes. Lloyd et al. [7], in contrast, investigated exoscopic versus microscopic visualization in simulated temporal bone surgery; these studies should not be conflated.

Exoscopic visualization also has limitations: the operative field depends on the camera line of sight, which may be obstructed by instruments or restricted in deep and narrow corridors. Camera repositioning and monitor orientation may require adjustment, and operating while viewing a screen entails adaptation. Surgical loupes provide direct vision and can be repositioned readily, but generally offer lower magnification and do not provide the same shared, highly magnified stereoscopic view of deep temporal bone recesses. Neither loupes nor conventional microscopy was used as a comparator in this patient.

The limitations of this report are substantial. A single case cannot establish the safety, effectiveness, or clinical superiority of ORBEYE™ compared with microscopy. No intraoperative microscopic comparator or standardized measures of image quality, ergonomics, or assistant experience were available; operative duration was recorded but cannot be interpreted comparatively. The presumed postoperative origin cannot be demonstrated from the history of myringoplasty alone because the original operative record was unavailable. Although histopathology confirmed cholesteatoma, the available operative observations cannot establish whether the pinpoint dural defect predated dissection. Intraoperative still photographs of individual operative steps could not be added; the operative video is identified as Supplementary Materials. Finally, the 18-month MRI and 24-month clinical findings are reassuring but do not replace longer surveillance of the reconstructed canal and mastoid cavity.

Further studies should evaluate exoscopic otologic surgery prospectively using standardized outcomes, including operative time, conversion to microscopy, visualization of critical anatomical structures, surgeon ergonomics, complication rates, audiological outcomes, and learning curves. Multicenter series would be particularly valuable for defining which otologic and lateral skull base procedures are most likely to benefit from exoscopic visualization.

## 5. Conclusions

This case illustrates the technical feasibility of a fully exoscopic, hearing-preserving approach to unusually extensive EACC with temporal bone erosion and dural exposure. Macroscopically complete removal and canal-wall reconstruction were achieved while preserving an intact tympanic membrane and middle-ear compartment. An intraoperative CSF leak occurred and was controlled during surgery, with no persistent postoperative leakage reported. The ORBEYE™ exoscope provided stereoscopic visualization throughout the operative stages, but this single case does not establish superiority or generalizable safety compared with conventional approaches. The presumed postoperative origin remains unproven, and further comparative studies are required.

## Figures and Tables

**Table 1:** Preoperative and postoperative outcomes.

Parameter	Preoperative (1 Month Before Surgery)	Postoperative (2 Months After Surgery)	Timing/Source
Air-conduction pure-tone average (dB HL)	31	17	500, 1000, 2000, 4000 Hz
Bone-conduction pure-tone average (dB HL)	12.5	8.5	500, 1000, 2000, 4000 Hz
Calculated air–bone gap (dB HL)	18.5	8.5	AC PTA minus BC PTA
Clinical EAC examination	Subtotal stenosis	Patent and epithelialized	24 months
Non-echo-planar DWI MRI	Preoperative diffusion restriction	No residual/recurrent EACC	18 months

Table note: AC, air conduction; BC, bone conduction; PTA, pure-tone average; ABG, air–bone gap; DWI, diffusion-weighted imaging; EAC, external auditory canal; EACC, external auditory canal cholesteatoma. The ABG values were calculated as the difference between the reported four-frequency AC and BC PTAs, ensuring internal numerical consistency.

## Data Availability

The information supporting this case report is contained in the article and its supplementary material. Further inquiries can be directed to the corresponding author.

## Supplementary Materials

The following supporting information can be downloaded at: https://www.mdpi.com/article/10.3390/jcm15218625/s1, Video S1, “Fully exoscopic surgical management of presumed postoperative external auditory canal cholesteatoma”.
